# A double *CYP27A1* gene mutation in spinal cerebrotendinous xanthomatosis in a patient presenting with spastic gait: a case report

**DOI:** 10.1186/s13256-024-04426-1

**Published:** 2024-07-11

**Authors:** Je Hong Min, Yoon Seob Kim, Myeong Jin Son, In Soo Joo

**Affiliations:** Department of Neurology, Ajou University School of Medicine, Ajou University Medical Center, 164, World Cup-Ro, Yeongtong-Gu, Suwon, Republic of Korea

**Keywords:** Cerebrotendinous xanthomatosis, CYP27A1, Spinal CTX, Myelopathy, Chenodeoxycholic acid

## Abstract

**Background:**

Cerebrotendinous xanthomatosis (CTX, OMIM #213700) is a rare inherited metabolic disease caused by the mutation in the *CYP27A1* gene. Spinal CTX is a rare clinical subgroup of CTX which lacks typical symptoms seen in classical CTX. Here we report a spinal CTX case revealed double mutation of *CYP27A1* gene.

**Case presentation:**

A 42-year-old Asian man visited our hospital with spastic gait started at 35. Physical examination showed bilateral masses on his Achilles tendons and were identified as xanthoma on ankle magnetic resonance imaging (MRI). Brain and spinal cord MRI revealed high signal lesions in bilateral cerebellar dentate nuclei and long tract lesions involving lateral corticospinal and gracile tracts. Gene analysis revealed double heterozygous mutation, c.223C > T (p. Gln75Ter) and c.1214G > A (p. Arg405Gln).

**Conclusions:**

We believe that novel mutation detected in our case might have a role in the pathomechanism in CTX. Moreover, spinal CTX should be considered in the patients only presenting with pyramidal symptoms, as CTX shows good prognosis in early treatment with chenodeoxycholic acid.

## Background

Cerebrotendinous xanthomatosis (CTX, OMIM #213700), is a rare autosomal recessive disorder of bile acid synthesis due to variants in the *CYP27A1* gene resulting in deficiency of sterol 27-hydroxylase, a key enzyme producing chenodeoxycholic acid (CDCA), which is a primary bile acid [1, 2]. Deficiency of this enzyme, in turn, produces precursor molecules: cholesterol and cholestenol. These molecules accumulate in many tissues, especially in the central nervous system (CNS), lens and tendons. Classical symptoms of CTX are infantile or childhood diarrhea, juvenile cataracts, tendon xanthomas and progressive neurological dysfunction such as mental retardation, seizures, cerebellar ataxias and pyramidal symptoms [2, 3]. However, there is another type of CTX, called “spinal CTX”. Its biochemical profiles are similar with classical CTX, but patients show slowly progressive symptoms of the corticospinal and dorsal column tracts without much of the classical symptoms mentioned above [4–6]. There are some reports of double mutation in classical CTX in Japan [7], in Chinese family [8] and also in South Korea mimicking behavioral variant frontotemporal dementia [9]. Here, we report a case of double mutation in spinal CTX only presenting with spastic gait.

## Case presentation

A 42-year-old Asian man was admitted to our hospital with slowly progressive gait difficulty over 7 years. Starting with minor discomfort in both legs, he complained of leg stiffness, slow gait, dragging of feet and imbalance. He denied any discomfort in his upper extremities.

**Fig. 1:**
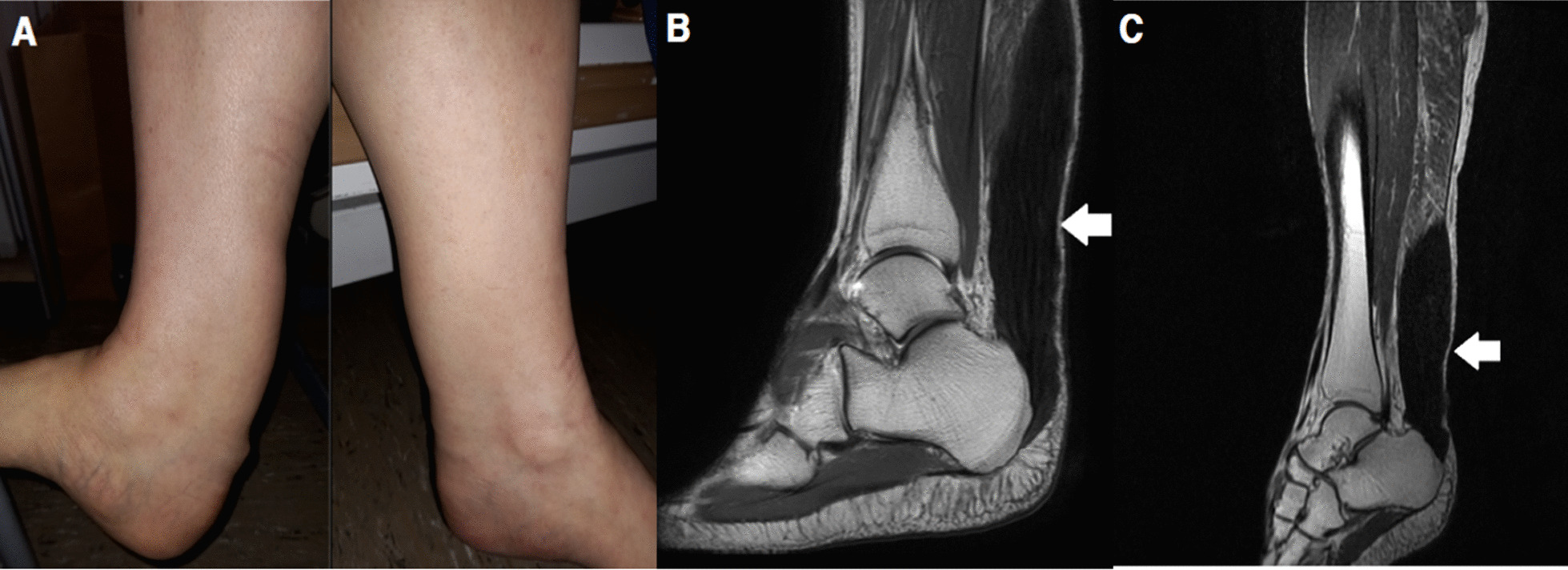
**A** Bilateral masses on the patient’s Achilles tendons. **B**, **C** Ankle MRI (T2-weighted image) revealed tendinous xanthomas on the patient’s both Achilles heels (white arrows), (**B**): left, (**C**): right

On neurological examination, he had intact mental and cranial nerve functions. His motor function and reflexes of the upper extremities were normal. The lower extremities showed normal power, but spasticity with brisk knee jerks and Babinski signs on both feet. His sensory function was normal. He had a narrow-based gait with short strides and lacked flexibility when avoiding obstacles but Romberg’s sign was negative. On physical examination, bilateral masses were found on his Achilles tendons (Fig. 1A).

He had no past medical history and denied any familial diseases related to his gait difficulty. However, his younger brother also had the masses in heels like him.

Laboratory tests showed no significant abnormalities apart from abnormally high triglyceride levels (788 mg/dL; normal 37–200 mg/dL). Total low-density lipoprotein and high-density lipoprotein were normal. Although no evidence of peripheral neuropathy was found in the nerve conduction study, his somatosensory evoked potentials and motor evoked potentials on his upper and lower extremities showed central conduction defects above the cervical level. Magnetic resonance imaging (MRI) scan of the ankle revealed enlarged Achilles tendons with diffuse low signal intensity on both sides, compatible with xanthoma (Fig. 1B, C). The Seoul Neuropsychological Screening Battery for screening cognitive function showed mild impairment. Screening for ophthalmologic complications was performed and revealed cataracts in his right eye.

**Fig. 2:**
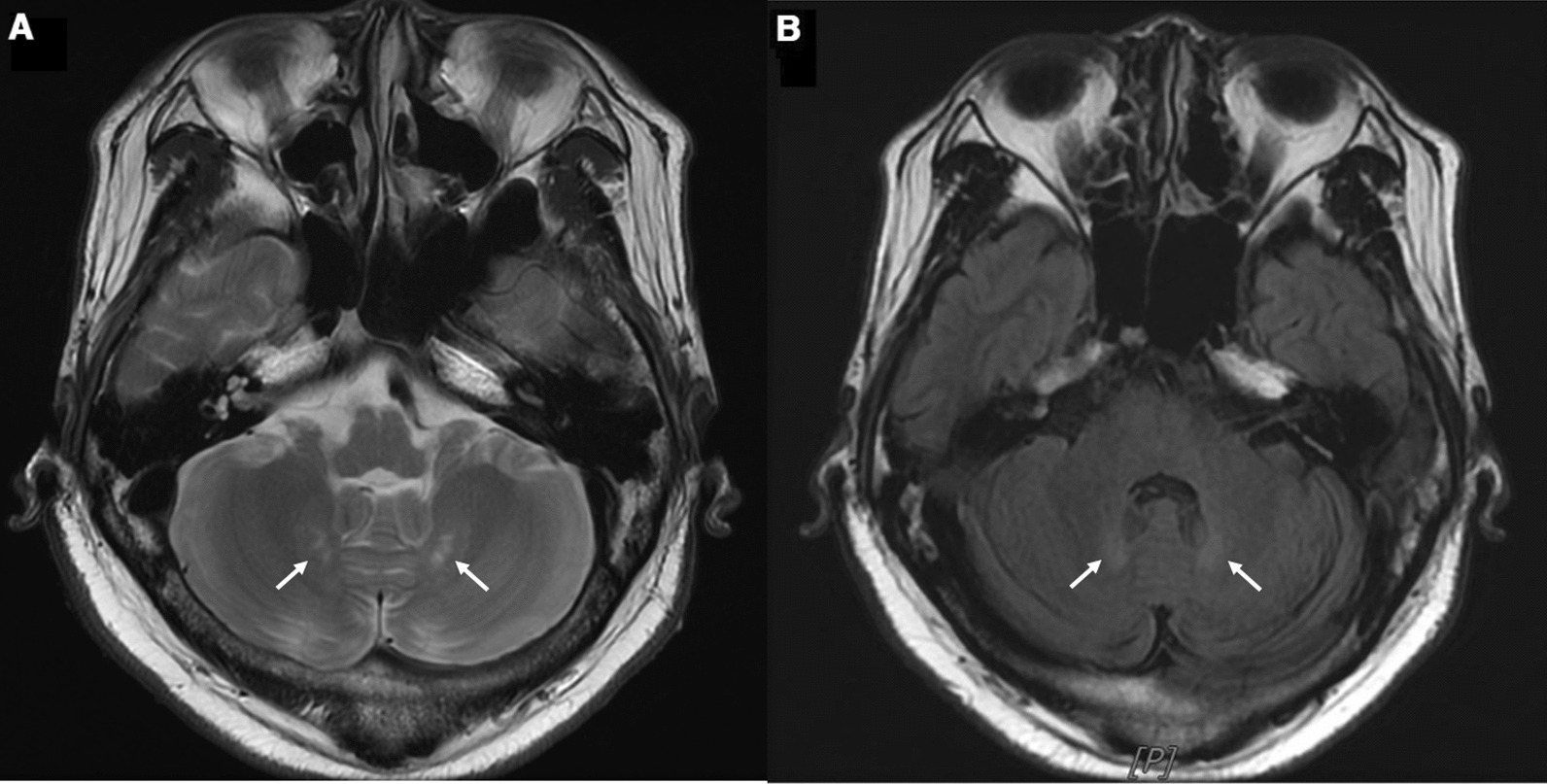
Ill-defined hyperintense lesions in bilateral cerebellar dentate nuclei were detected (white arrows) on axial T2-weighted image (**A**) and FLAIR image (**B**) of the brain

**Fig. 3:**
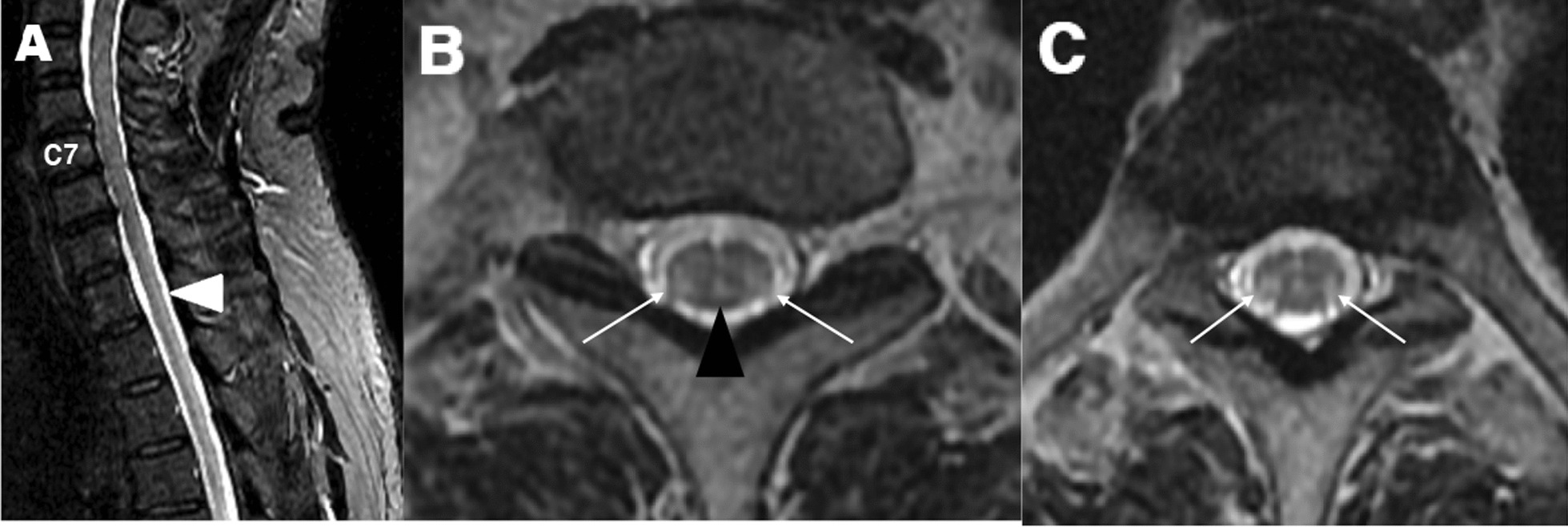
Spinal cord MRI of the patient. Sagittal T2 images showed longitudinal T2 hyperintensity of the posterior columns at upper thoracic level (white arrowhead) (**A**) and axial T2 images confirmed bilateral, symmetric high signal intensities of lateral corticospinal tracts (**B, C**, white arrows) and the gracile tracts (**B**, black arrowhead) at upper thoracic level without gadolinium enhancement

Brain MRI revealed increased signal intensity in bilateral cerebellar dentate nucleus on T2-weighted and fluid-attenuated inversion recovery (FLAIR) images (Fig. 2A, B). Spinal cord MRI showed long hyperintense lesions involving lateral corticospinal and gracile tracts from the lower cervical to upper thoracic level on T2-weighted images (Fig. 3).

Genetic analysis was performed with next generation sequencing (NGS) panel for spastic paraplegia and two heterogeneous pathogenic variants of the *CYP27A1* gene were identified. One was a nonsense mutation of c.223C > T, which is predicted to generate premature stop codon of p. Gln75Ter, and the other was a missense mutation of c.1214G > A in exon 7, which is predicted to alter the amino acid of p. Arg405Gln. According to the 2015 American College of Medical Genetics and Genomics guidelines, c.223C > T can be classified as a ‘likely pathogenic variant’ and c.1214G > A can be classified as a ‘pathogenic variant’.

We made a diagnosis of CTX and the patient was treated with chenodeoxycholic acid (CDCA) and ursodeoxycholic acid (UDCA) combination at 750 mg/day and our patient was discharged with good general condition.

## Discussion

The classical form of CTX has characteristic features such as tendon xanthomas, childhood-onset diarrhea, juvenile cataract and neurological dysfunction. However, spinal CTX has been rarely reported, so it is often initially misdiagnosed because of its lack of classical symptoms. In our case, the patient visited our hospital only with spastic gait, which could be confused with other spinal cord diseases such as hereditary spastic paraparesis. Typical MRI findings of spinal CTX show longitudinal white matter lesions in the lateral corticospinal and gracile tracts at the spinal cord level [1, 4, 5] and these findings can be accompanied by cerebral lesions [6, 10]. Pathologically, extensive and symmetric loss of myelin and axonal loss related to gliosis and perivascular accumulation of macrophages are present, especially in lateral corticospinal tracts and gracile tracts [1]. As genetic confirmation has more important value than serum biochemical markers in diagnosing CTX [11], we could diagnose CTX with the detection of *CYP27A1* pathogenic variants in this case, without checking serum cholestenol levels.

In this patient, two heterozygotic mutations were revealed in the *CYP27A1* gene, c.1214G > A (p. Arg405Gln) and c.223C > T (p. Gln75Ter). The c.1214G > A (p. Arg405Gln) mutation is well-known for its pathogenicity based on the report that its residues are located near the functional domain of sterol 27-hydroxylase and affect the enzyme activity of sterol 27-hydroxylase [12]. Although the c.1214G > A (p. Arg405Gln) allele is commonly reported in spinal CTX as well as classical CTX [3, 13] and its function is well known for affecting enzymatic activity, there is no evidence of phenotypic preference in either classical or spinal forms of CTX [14]. The other mutation, c.223C > T (p. Gln75Ter), has not been reported in CTX but was predicted to be a likely pathogenic variant in in-silico analysis, by using bioinformatics tools such as SIFT (https://sift.bii.a-star.edu.sg/), PolyPhen2 (http://genetics.bwh.harvard.edu/pph2/) and Mutation Tester (http://www.mutationtester.org/).

Atallah *et al.* [5] had reviewed 33 patients of spinal CTX cases, and most patients had accompanied with cataracts (78%) and chronic diarrhea (65%). Moreover, almost half of the patients (48%) had dorsal column signs and brain MRI revealed cerebral white matter lesions with same percentage. But in our case, patient had only pyramidal symptom with incidentally diagnosed cataracts without cerebral white matter change in brain imaging study. As there is a case report of CTX with double mutation in the *CYP27A1* gene [7, 9], we believe that c.223C > T allele of this gene may have a role in the spinal CTX dominant with pyramidal signs but future study is needed to determine the relevance of pathogenicity in this disease.

Because the spinal form of CTX is rare and lacks typical symptoms observed in classical CTX [10, 13], we should distinguish this disease from other CNS disorders, such as demyelinating, infectious, metabolic and neurodegenerative disorders [10, 15]. Therefore, it is difficult to diagnose CTX only with pyramidal symptoms, as in our case.

However, because CTX is a genetic disease related to sterol 27-hydroxylase enzyme deficiency, treatment with CDCA, which is the ingredient of bile acid, is effective in normalizing serum biochemical abnormalities. Early treatment initiation can lead to a good prognosis and prevent further neurological disability [16], so early diagnosis of CTX is very important for the patient’s clinical course.

## Conclusions

We reported double mutation of *CYP27A1* gene in spinal CTX with harboring a novel mutation. Moreover, our case highlights the difficulty of considering CTX in patients presenting only with pyramidal symptoms and emphasizes the importance of early consideration of CTX in this condition due to benefits from early treatment.

## Data Availability

Not applicable.

## Abbreviations

CTX: Cerebrotendinous xanthomatosis
CDCA: Chenodeoxycholic acid
UDCA: Ursodeoxycholic acid
